# The Genetics of Risk Aversion: A Systematic Review

**DOI:** 10.3390/ijerph192114307

**Published:** 2022-11-02

**Authors:** Francisco Molins, Fatmanur Sahin, Miguel Ángel Serrano

**Affiliations:** Department of Psychobiology, Universitat de València, Av. Blasco Ibáñez, 13, 46010 Valencia, Spain

**Keywords:** risk aversion, loss aversion, genetics, polymorphism, decision-making, emotions, serotonin, dopamine

## Abstract

Risk and loss aversion are phenomena with an important influence on decision-making, especially in economic contexts. At present, it remains unclear whether both are related, as well as whether they could have an emotional origin. The objective of this review, following the PRISMA statements, is to find consistencies in the genetic bases of risk and loss aversion with the aim of understanding their nature and shedding light on the above issues. A total of 23 empirical research met the inclusion criteria and were included from PubMed and ScienceDirect. All of them reported genetic measures from human samples and studied risk and loss aversion within an economic framework. The results for risk aversion, although with many limitations, attributed mainly to their heterogeneity and the lack of control in the studies, point to the implication of multiple polymorphisms related to the regulation of the serotonergic and dopaminergic pathways. In general, studies found the highest levels of risk aversion were associated with alleles that are linked to lower (higher) sensitivity or levels of dopamine (serotonin). For loss aversion, the scarcity of results prevents us from drawing clear conclusions, although the limited evidence seems to point in the same direction as for risk aversion. Therefore, it seems that risk aversion could have a stable genetical base which, in turn, is closely linked to emotions, but more research is needed to answer whether this phenomenon is related to loss aversion, as well as if the latter could also have an emotional origin. We also provide recommendations for future studies on genetics and economic behavior.

## 1. Introduction

According to classical economics, a rational decision-maker would follow the principle of optimization and should always choose the prospect (decision option) with the highest utility [1,2]. Mathematically, “the utility of a monetary gamble is a weighted average, where each possible outcome is weighted by its probability of occurrence” [3], p. 341. However, consider the following example: you must choose between prospect A, which offers an 85% chance to gain 1000 EUR (with a 15% chance to gain nothing), or prospect B: a certain chance of obtaining 800 EUR. In this example, prospect A would have a utility of 850 EUR (0.85 × 1000 + 0.15 × 0), while prospect B would have a utility of 800 EUR (1 × 800). Thus, a rational decision-maker should choose option A. However, it has been demonstrated that most people prefer the safe option despite its lower utility, thus avoiding the risky option [3]. This preference for a sure outcome over a gamble with higher or equal utility is called risk-aversion [3,4] and could constitute an anomaly regarding economic theory.

Bernoulli [5,6] provided a logical explanation whereby risk aversion could be saccommodated within the classical economics assumptions: people would not evaluate prospects based on the objective outcome, but on the subjective value of that outcome. Furthermore, Bernoulli [6] proposed that this subjective or expected utility follows a concave function, where the difference between the utilities of 200 EUR and 100 EUR is greater than the difference between the utilities of 1200 EUR and 1100 EUR. Therefore, the expected utility attached to a gain of 800 EUR would be higher than the expected utility of 1000 EUR with a probability of 80%, even if both prospects had the same (mathematic) utility. Thus, risk aversion may still have a place as a rational behavior within the established framework. However, when Kahneman and Tversky [7] developed the prospect theory, they reported anomalies that could not be easily accounted for under rational assumption. They found that the tendency to be risk-averse is reversed, turning people into risk-seekers, when decisions are framed in terms of loss. This phenomenon was called framing effect [3,4,8] and is considered an anomaly since it violates one of the main axioms of rationality: the invariance axiom [3,9], whereby the preference between prospects should not depend on the way they are described [3,10].

An explanation to this anomaly is that human beings do not always act rationally nor follow the strategic decision-making proposed by the classical models [11]. Many of our decisions are fast and frugal, influenced by cognitive limitations and biases [1,9,12]. In this line, both risk aversion and framing effect could be built on the basis of a more basic, automatic, and emotional phenomenon [4,13]: the loss aversion bias [9,14]. In the words of Rabin and Thaler [4], p. 226, “loss aversion is the tendency to feel the pain of a loss more acutely than the pleasure of an equal-sized gain”. This phenomenon could account for the framing effect, since people would not risk losing if they could choose a certain gain but would prefer to risk rather than accept a certain loss.

There is still a debate on these issues and many economists are trying to accommodate anomalies within the already established, orthodox model to demonstrate that their statements are valid. However, neuroscience is providing increasing evidence that, in fact, risk aversion, framing effect, and loss aversion are part of the same reality, sharing a neural bases and producing similar physiological responses [15,16,17,18,19,20]. Furthermore, such evidence would give strength to the argument that these phenomena are automatic and emotional in nature, rather than the result of thoughtful, planned thinking that fits into the rationality axioms. Thus, their neural bases are composed by structures such as the amygdala, insula or striatum [15,17], regions that constitute fundamental nodes in the salience network and the brain’s reward system, which, in turn, are key to the production of emotional responses [21,22,23,24,25].

Moreover, risk aversion is not exempt from individual variability. This heterogeneity seems to depend on multiple factors such as age, gender or level of education, although these only predict a small fraction of the variance in risk-taking [26]. Multiple studies with twins (homozygous and heterozygous) have shown that risk aversion could depend between 20% [27,28] and almost 60% [29] on genetic variations. In fact, more specifically, studies such as Kuhnen & Chiao [30] have located some of these variations, indicating that certain polymorphisms, such as DRD4 or 5-HTTLPR, which are related, respectively to the differential expression of dopamine and serotonin receptors, are also associated with different levels of risk-taking. A polymorphism denotes that the same gene can take different forms or alleles, which will translate into different phenotypes or expressions of that gene. Depending on the form the gene takes, risk aversion is manifested to a greater or lesser extent. Moreover, it has been also found a relationship between the different alleles of the 5-HTTLPR or DRD2 (similar to DRD4) polymorphisms and the different levels of loss aversion [31,32]. As can be seen, these studies also point to the link between these phenomena, as well as to their emotional nature, given that serotonin and dopamine are the main neurotransmitters involved in processes of emotional regulation and response to threats and rewards [33,34,35]. However, most of these studies are recent and show heterogeneity in both methods and results.

The present study carries out a systematic review covering the published literature on genetics and risk aversion (including framing effect and loss aversion given their possibly close links), with the aim of clarifying the debate between the prospect theory and classical economics by shedding light on the question of whether risk aversion is a behavior that fits within the rationality assumptions or, on the contrary, has an emotional origin connected to loss aversion. Our first hypothesis (hypothesis 1) states that there would be a consistent genetic base for risk aversion. On the other hand, (hypothesis 2) states that risk aversion and loss aversion would have a common genetic origin, with both phenomena being connected. Finally, (hypothesis 3) this origin would be related to our affective system.

## 2. Materials and Methods

**Figure 1:**
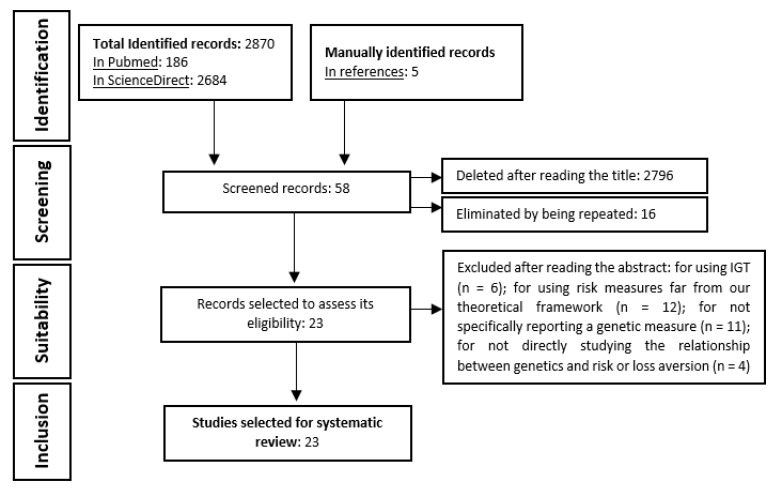
Revision flowchart.

A systematic review of the genetic bases of risk aversion (also including related phenomena: framing effect and loss aversion) has been carried out, following the guidelines of the PRISMA declaration [36] for the correct performance of systematic reviews. The elaboration phases will be detailed below (and see Figure 1).

### 2.1. First Searches

The first searches were conducted in November 2021. To obtain an overview of the available results, as well as to see which terms worked best, Boolean operators AND and OR were used, trying different combinations of the following terms: ‘loss aversion’, ‘losses aversion’, ‘risk aversion’, ‘risk avoidance’, ‘risk seeking’ ‘risk taking’, ‘framing effect’, ‘genetic’, ‘genetics’, ‘genetic bases’, ‘gene’, ‘genes’, and ‘polymorphism’. These searches yielded a considerable number of results, many repeated or of little utility, but provided a preview and allowed for us to check that no previous review had been carried out on the topic. As the results of Scopus were the scarcest and did not provide any new studies, they was removed from the systematic search.

### 2.2. Systematic Search

The systematic search was conducted again in November 2021, in PubMed and ScienceDirect. The combination of terms that yielded the best results in both search engines was as follows: ((“loss aversion”) OR (“losses aversion”) OR (“risk aversion”) OR (“risk taking”) OR (“risk avoidance”) OR (“risk seeking”) OR (‘framing effect’)) AND (“genetic”). Specifically, 186 results were obtained in PubMed and 2684 in ScienceDirect. Before selecting articles, inclusion and exclusion criteria were defined.

#### 2.2.1. Inclusion Criteria

− Empirical research and not revisions, single case studies, theoretical frameworks, books or manuals.
− That specifically and directly report genetic measures (e.g., how different alleles of a polymorphism are related to risk aversion levels).
− That use human samples.
− That study risk aversion, framing effect, and loss aversion understood within the economic framework.
− That measure risk aversion, framing effect, and loss aversion with economic tasks.

#### 2.2.2. Exclusion Criteria

− Those that approach risk aversion, framing effect, or loss aversion from a far perspective from economics (e.g., risk taking related to sexual risk behavior, gambling disorder or substance abuse).
− Those who use non-economic tasks or use self-reports of risk preferences (i.e., questionnaires).
− Those that are studied in twins, or in terms of biological aspects, but do not report specific genetic measures.
− Those who study risk aversion with Iowa Gambling Task (IGT) [37]. As recently noted Lin et al. [38], the measure of risk inferred from this task is not as direct and is influenced by other factors, such as reward-learning or the sensitivity to feedbacks, which may make it difficult to interpret results in this review. In addition, IGT can be considered a decision-making measure in the face of ambiguity rather than risk, which is a different approach to this study [39].

According to the established criteria, and after only reading the title, 58 articles were considered as adequate (after eliminating sixteen duplicates between the two databases). The abstract was read and, from the identified group, 40 were discarded: for using IGT (*n* = 6), for using risk measures far from our theoretical framework (*n* = 12), for not specifically reporting a genetic measure (*n* = 11) and for not directly studying the relationship between genetics and risk aversion, framing effect, or loss aversion (*n* = 4). The remaining 18 articles comply with the criteria described above.

### 2.3. Manual Search

After an in-depth reading of the 18 selected studies and based on their references, five new articles that had not appeared in the databases were included. All referred to risk aversion (although three of them approached risk aversion trough the framing effect). Finally, Google Scholar was used with different combinations of the mentioned search terms to check whether any article could have been left out. These searches did not reveal any new relevant studies.

Finally, the systematic review includes a total of 23 empirical articles, all of them written in the English language. Nineteen referring to risk aversion; 2 to both risk aversion and loss aversion, and 3 only to the latter. In addition, most used gambling tasks, investment tasks or similar, with the exception of 4, which used the Balloon Analogue Risk Task (BART) [40], a task that is far from the others in terms of dynamics, but we decided to include it because it provides a direct indicator of risk-aversion that is comparable to the other tasks.

## 3. Results

The results are summarized in Table 1 and Table 2: the first one for risk aversion (including framing effect) and the second one for loss aversion. This division was made to facilitate the comparison between both phenomena. The explanation of the results follows the same division. Moreover, the results within each block were organized based on the genetic measure used, with all those referring to the same measure being reported together (e.g., to the same polymorphism).

**Figure 2:**
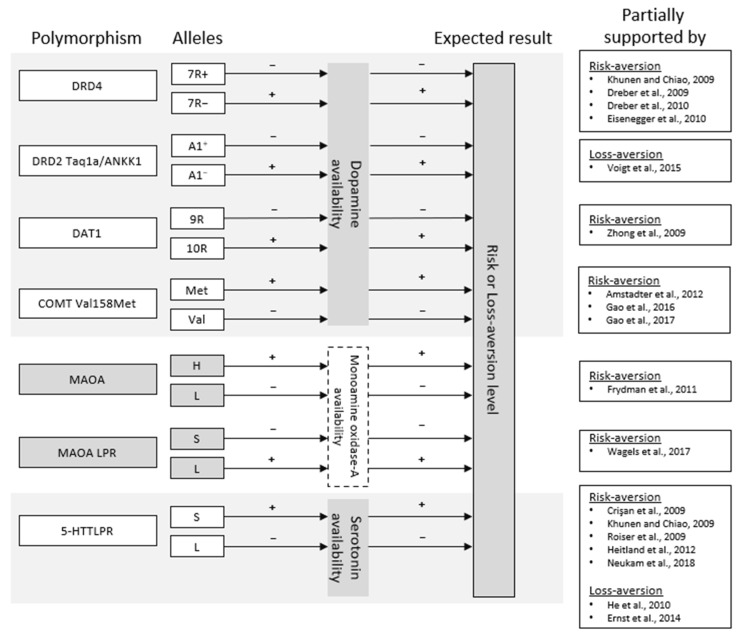
Scheme of the connection between the alleles of the different polymorphisms, the level of neurotransmission and the expected behavioral outcome (risk or loss aversion). On the right, the articles that support, at least partially, the expected result are shown, indicating if the support is found in risk or loss aversion.

An article (only for risk aversion) [26], comprehensively addressed the possible association between more than 2 million single nucleotide polymorphisms (SNPs) extracted from the U.S. Health and Retirement Study (HRS), and the risk-aversion level, measured with hypothetical gambles on lifetime income, in a sample of 10,455 adults. The remaining articles included in the review dealt with more specific genetic measures, known as “candidate-gene” studies [41]. They studied specific genetic markers (one or several genes, or regions thereof), since, based on prior knowledge of their biological functions, these could be related to the phenotype of interest (in this case, risk or loss aversion). Thus, we could compare whether the allele “x” that can adopt a gene, which, in turn, is related, for example, to higher serotonin levels, also implies a higher risk or loss aversion level with respect to the allele “y” of the same gene. Figure 2 was developed to facilitate reading.

Finally, to avoid over- or under-representing the role that an allele plays in the relationship between genes and risk or loss aversion, possible confusing variables must be controlled. Some examples could be a certain level of impulsiveness, a sensation-seeking personality or suffering from Reward Deficiency Syndrome (RDS). The health and behavioral profile of the participants was included in the results (see Table 1 and Table 2). However, as can be noted, few articles considered these aspects beyond “including a healthy sample” (without psychopathologies, illness, and medication/drug consumption). Only two articles conducted a more exhaustive screening, but they did so when choosing the sample and did not control for these variables when contrasting the level of bias as a function of the gene alleles (e.g., covariating the level of impulsivity).

### 3.1. Risk-Aversion

#### 3.1.1. Single Nucleotide Polymorphisms

According to Harrati [26], none of the multiple SNPs included in the study revealed a significant causal effect on risk aversion, pointing to greater phenotype complexity, as well as their possible polygenic origin. No other measures of health or behavior were considered in the study.

#### 3.1.2. SLC6A4 Candidate Gene

One of the most-studied candidate genes in relation to risk aversion was the SLC6A4, the only gene that encodes the serotonin transporters (5-HTT), responsible for the reuptake of serotonin from the synaptic cleft [33]. In the SLC6A4 regulatory region, there is a polymorphism, known as linked polymorphic region (5-HTTLPR). This involves an insertion or deletion of 44 nucleotide pairs (or 44-base pair), which may result in a short (s) or a long (l) allele. The transcriptional activity of 5-HTT will be modulated by the form this polymorphism takes. Thus, if the allele s is present, less 5-HTT will be expressed and its function will be reduced with respect to allele l, implying limited serotonin reuptake and greater serotonin availability in the synaptic cleft. Many studies that included in this review examined whether risk aversion varied with these alleles. In general, when analyzing the genotype for 5-HTTLPR, a distinction was made between homozygous individuals, whose two alleles are short or long (s/s or l/l), and heterozygous, with each allele in one form (s/l).

Genotyping 32 healthy participants (23 women), the study by Crişan et al. [33] found that those who carry even one s allele of 5-HTTLPR made fewer attempts to inflate the balloon at BART than homozygous l/l. That is, s-carriers showed greater risk aversion. However, s-carriers also showed a higher anxiety trait measured with STAI and EMAS. This trait was studied in parallel, but was not controlled to extract the above result.

A similar result can be seen in Heitland et al. [50], where s-carriers also had higher risk aversion, but only when they were in a gain context, using a gambling task. It should be noted that only healthy women participated in this study (*n* = 60).

With 65 participants (48 women), Kuhnen & Chiao [30] also showed similar results to Crişan et al. [33], this time using an investment task. However, here it was specified that only homozygous s/s, with respect to l/l or s/l, and not only those carrying a short allele, had a higher risk aversion.

Neukam et al. [56], with 577 healthy participants and an economic decision-making task, indicated the opposite result, reporting that s/s-carriers presented greater risk-seeking than s/l or l/l, but only in a loss context. No other significant result was found for the gain context.

Focusing specifically on framing effect, Roiser et al. [43] pointed out that, after genotyping 30 healthy participants and using a gambling task, homozygotes s/s showed the highest framing effect with respect to the s/l or l/l. That is, s/s-carriers risked significantly more in the loss contexts than in the gain contexts, a difference that was not observed with the s/l or l/l-carriers. The greater framing-effect was also accompanied by increased amygdalin activity. These results were extracted, controlling for personality traits, impulsiveness, and state-trait anxiety.

Turning again to Crişan et al. [33], with the same sample, but using a gambling task, the authors also studied framing effect. Again, it seems that the short allele favored a higher framing-effect; however, on this occasion, a homozygous genotype (s/s) did not seem necessary, and was enough to transport a single allele s.

On the other hand, in Frydman et al. [47], with a male sample (*n* = 83), using a gambling task; in Anderson et al. [52], with 174 inverters; or Zhong et al. [44] with 350 participants (188 women), using a multiple price list design (a task with a dynamic similar to a gambling task), no relationship was foud between the different alleles of 5-HTTLPR and the risk aversion measure. However, the latest study [49], reported that l-carriers tended to tolerate risk better (less risk aversion) in the context of losses than s-carriers. None of these studies reported data on health or other behavioral aspects of the sample.

#### 3.1.3. DRD4 Candidate Gene

The other candidate gene that, together with 5-HTTLPR, appeared more frequently in the articles of this review, was the dopamine receptor D4 gene (DRD4), involved in the regulation of the dopaminergic system [18]. There is a region in DRD4 where several repeats of a DNA base pairs sequence can appear. Each individual of the same species can present a different number of repetitions of this sequence, which is most frequent between 2 and 11 repetitions [49]. When the genotype for this gene is extracted, it is usually dichotomized. If both alleles of the gene have fewer than 7 repeats, it is assigned the term 7R−; if either allele has more than 7 repeats, then it is assigned 7R+. The presence of 7R+ is usually associated with a lower effectiveness in the receptor-ligand junction [18]; in other words, this means that the dopamine receptor needs more dopamine to produce the effect which, in the case of individuals with 7R−, would be achieved with less dopamine. Again, an attempt has been made to study whether this polymorphism can be related to different levels of risk aversion.

First, this gene appeared in the Kuhnen and Chiao study [30] mentioned in 5-HTTLPR. Thus, with the same sample of 65 participants (48 women) and using an investment task, it was observed that the 7R+ carriers assumed more risks (lower risk aversion) than the 7R− carriers.

On the other hand, Dreber et al. [18], found the same result using the same task (investment task), but including only healthy men in their sample (*n* = 98).

With a larger sample (237 participants) and again including women, but this time, not controlling for health or behavioral variables, Dreber et al. [49] used the same task again, and also added the card game bridge, similar to a gambling task, but with the more playful context of card games. Here, the same result found in previous studies was reported (lower risk aversion in 7R+ carriers), in both tasks, but only in men. Women showed no difference in risk aversion between 7R+ and 7R−.

The remaining articles referring to DRD4 found no differences in risk aversion depending on their alleles. Frydman et al. [47] included 83 male participants and used a gambling task. In Dreber et al. [45], 125 participants (8% women) were included, and an investment task was used. In Anderson et al. [52], 174 investors were included and a task similar to a gambling task was used. In Muda et al. [55], 113 investors and 104 non-investors were included and the Holt–Laury test was used. Finally, in Eisenegger et al. [46], 205 healthy male participants were included and a gambling task was used again. Although no study reported significant relationships between DRD4 and risk aversion, the latest study used a dopamine administration protocol to compare whether, after this, the alleles of DRD4 behaved differently. In this sense, the 7R+ carriers seemed to have a lower risk aversion than the 7R− after the administration of dopamine. Except for this last study, the other three did not even control for whether the participants were healthy.

#### 3.1.4. ANKK1 Candidate-Gene

Another gene involved in the regulation of the dopaminergic system was the Ankyrin repeat and kinase domain containing 1 (ANKK1), linked to the gene of the dopaminergic receptor D2 (DRD2) and affecting the expression and functioning of this receptor [32]. ANKK1 has a polymorphism known as Taq1a, which can have two different forms on its alleles: A1 or A2. These give rise to three possible combinations: A1/A1, A1/A2 and A2/A2, although it is usually dichotomized in A1+ versus A1-, depending on whether the A1 allele is present or not. The A1 allele is usually associated with a less striatal D2/3 receptor-binding which, like DRD4, could indicate that more dopamine is required to produce proportional effects to those which, without this allele, would be produced with lower amounts of dopamine.

For risk aversion, only one study addressed this polymorphism. Dreber et al. [18], already mentioned in DRD4, with 98 healthy males and using an investment task, found no differences in risk aversion between the A1+ and A1- genotypes.

#### 3.1.5. MAOA Candidate Gene

The next candidate gene was the MAOA gene that codes for the enzyme MAOA (monoamine oxidase-A). This enzyme is involved in the metabolism of serotonin and dopamine [49]. The MAOA gene also has a polymorphism. As in 5-HTTLPR, there is a base-pairs sequence that is repeated and in which, depending on the number of repetitions, the alleles are considered to be either low active (3 or 5 repetitions, MAOA-L) or highly active (3.5 or 4 repetitions, MAOA-H) [49]. MAOA-L is related to a lower transcriptional activity of the MAOA gene and, therefore, a lower amount of the MAOA enzyme. Both alleles were studied in relation to risk aversion.

Frydman et al. [47], with the sample and decisional task described above, found a relationship between the MAOA-L allele and a higher probability of assuming risks (lower risk aversion).

However, turning to Dreber et al. [49], the result was the absence of a relationship and differences between MAOA-L or MAOA-H and risk aversion.

In addition to the polymorphism described for MAOA, this gene may present another polymorphism in its promoter region, known as MAOA LPR (as in 5-HTTLPR, the acronym stands for linked polymorphic region). This depends on the number of repetitions of a sequence of 30 base pairs, which may result in the short (allele s) or long (allele l) version [54]. The allele s was related to the lower coding and effectiveness of the above MAOA.

In relation to this polymorphism, Wagels et al. [54] studied risk-taking in 105 healthy male participants using BART. They found that MAOA s-carriers did not differ from MAOA l-carriers in assuming risks in this task; however, they showed greater latency times when avoiding risk. These results were found to measure and control for anxiety and aggressiveness traits.

#### 3.1.6. SLC6A3 Candidate-Gene

Other candidate-gene was SLC6A3, which participates in dopaminergic pathways by encoding dopamine transporters (DAT), and which, as in SLC6A4 (for 5-HTT), shows a polymorphism known as DAT1, depending on the number of repetitions of a sequence of 44 base pairs. Although these repetitions can oscillate between 3 and 13 times, the most common is to find 9 or 10 repetitions [51]. Thus, we speak of allele 9R and 10R or, depending on the presence or absence of allele 9R: 9R+ and 9R−. The 9R allele is associated with a lower expression of DAT in the striatum compared to the 10R allele, which translates into greater availability of dopamine in the synaptic clefts of this region [53].

The study of Mata et al. [51], with 322 healthy participants (234 women), and using BART, found that those genotypes that present at least one 10R allele made more attempts to inflate the balloon, implying lower risk aversion, compared to homozygous for 9R.

On the other hand, Heitland et al. [50], also studied, using the same task (a gambling task), the relationship between risk aversion and DAT1. The authors reported the absence of a relationship between any of the alleles of DAT1 and the attitude towards risk.

Finally, the study by Zhong et al. [44] included the DAT1 polymorphism approach. However, here, the authors maintained a different theoretical approach, affirming that it was the 9R allele (and not the 10R), which would imply a lower level of dopamine in the striatal synapses. Thus, when studying how this polymorphism influenced risk-aversion, they reported a relationship between the 9R−carriers and lower risk aversion levels.

#### 3.1.7. COMT Candidate-Gene

Another candidate gene that also appeared with some frequency is the Catechol-o-methyltransferase (COMT) gene that encodes the COMT enzyme, one of the main ones responsible for the degradation of dopamine. This gene presents a polymorphism (COMT Val158Met) in a specific base-pair where, depending on whether guanine or adenine is present, the gene will contain valine (Val) or methionine (Met), respectively [54]. Thus, homozygous (Met/Met or Val/Val) or heterozygous (Met/Val) genotypes may occur. Met alleles are usually associated with lower COMT activity and, therefore, lower dopamine degradation; in other words, higher dopamine availability, especially in the prefrontal cortex [56].

In the study by Gao et al. [42], employing a sample of 111 healthy students (36% female) and focusing on framing effect with a gambling task, they found that those genotypes that carried at least one Met allele were predisposed to assume more risks in loss contexts than in gain contexts; that is, a greater framing effect with respect to homozygous for the allele Val. Furthermore, this relationship between genes and bias seemed to be mediated by resting-state functional connectivity between orbitofrontal cortex and bilateral amygdala.

However, in both Heitland et al. [50], as described when talking about 5-HTTLPR and DAT1, and the study by Roe et al. [58], which used a gambling task to measure the attitude towards risk in 67 participants (29 women), no relationship was found between COMT Val158Met polymorphism and the different risk-aversion levels. In the last study sample, most were healthy volunteers (*n* = 55); however, 12 participants diagnosed with depression and bipolar disorder were also included. These participants were not monitored when the results were extracted.

Finally, Amstadter et al. [48], with a sample of 223 healthy children (44.4% female) and using BART, reported a result that could be contradictory to that of Gao et al. [42]. Here, it was shown how the Met allele was associated with lower risk aversion compared to homozygous for the Val allele; however, this result only occurred in women, and was not found in relationships in men, suggesting that the effect of sex could act as a modulator.

#### 3.1.8. Multiple Candidate Genes at Once

Gao et al. [53] addressed framing effect with a mid-way approach between candidate-genes and the comprehensive study of SNPs. Here, the possible relationship between framing effect and multiple polymorphisms (of 26 different genes) was widely studied, but all of them can be considered candidate genes because of their influence on dopaminergic and serotonergic pathways, which, as we have seen in previous studies, seem to be involved in the differential expression of risk aversion and framing effect. These polymorphisms included all those mentioned above (except DRD4) and other new ones, such as the vesicular monoamine transporter (VMAT2), tryptophan 5-hydroxylase (TPH 1 and TPH2) or tyrosine hydroxylase (TH), among others. The sample comprised 1582 healthy students (80.1% women) and the task used to measure the framing-effect was a classic gambling task adapted to a pencil-and-paper format to collect a larger sample. In a broad way, the analyses carried out by Gao et al. [53] revealed that the genetic variations in the genes SLC6A4 and COMT, related, respectively, to the serotonergic and dopaminergic pathways, influenced individual differences in framing effect. In addition, the importance of the DDC gene (aromatic-L-amino-acid decarboxylase), involved in the two routes mentioned above, was also highlighted.

### 3.2. Loss-Aversion

The results found in the scientific literature for loss aversion were very scarce in relation to risk aversion (only five articles). All of them focused on candidate-gene polymorphisms that appeared in the previous block, except one. The most repeated (in 4 of the 5 articles) was 5-HTTLPR polymorphism (see previous section for an explanation).

5-HTTLPR was addressed for loss aversion in two of the studies already mentioned for risk aversion: Anderson et al. [52] y Neukam et al. [56] (see previous section for details of the sample). The first study measured loss aversion with a survey that raised hypothetical choices, in line with a typical mixed gamble task, and the second study used the latter task. However, no relationship between loss-aversion and any of the alleles of 5-HTTLPR (s or l, both homozygous and heterozygous) was found.

Something similar occurred in Ernst et al. [57], who used a mixed gamble task to study loss aversion in a sample of 66 adolescents, of whom 27 had an anxiety disorder. Therefore, the control group of healthy adolescents did not show any difference in loss aversion according to their genotype. However, something different occurred when we focus on patients with anxiety, showing the lowest level of loss aversion by those who were homozygous for allele l. No differences were found with respect to the control group for homozygous s/s.

On the other hand, the study by He et al. [31], also using a mixed gamble in a sample of 572 healthy participants (312 women), found differences between the alleles for loss aversion. Specifically, homozygous s/s had greater loss aversion than s/l or l/l. In addition, these effects were more clear in men than in women (although they were statistically significant in both sexes).

Another polymorphism that was addressed, although only in one study, was DRD4 (see more details in the risk-aversion section). Specifically, this was included in the aforementioned study by Anderson et al. [52], which suggested that different alleles of DRD4 (7R+ and 7R−) were not related to loss aversion.

The latest study included in the review was Voigt et al. [32], who focused on the relationship between loss aversion, measured with a mixed gamble and a sample of 143 participants (114 women) and two polymorphisms. The first, DRD2, was already explained in the risk aversion section; the second was BDNF Val66Met. This is found in the BDNF gene that encodes for the brain-derived neurotrophic factor (BDNF). As with the polymorphism COMT Val158Met, two genetic variants can be presented, Val and Met, depending on whether it contains valine or methionine. Again, the Met allele is associated with a lower effectiveness; in this case, in the production of BDNF [32]. Each of these polymorphisms separately (DRD2 and BDNF Val158Met) did not present significant effects on loss aversion; however, when its interaction was studied, it was found that those who have at least one Met allele in BDNF Val158Met and one A1 allele in DRD2 in their genotype displayed the lowest level of loss aversion.

## 4. Discussion

The main objective of this review was to synthesize the genetic bases available in the scientific literature on risk aversion and close-related phenomena: framing effect and loss aversion. Although the results are not exempt from nuances and limitations, they pointed towards a series of genetic polymorphisms, all of them involved in dopaminergic and serotonergic neurotransmission pathways and which, depending on the alleles they acquired, seem to modulate the above-mentioned behaviors. This is especially remarkable for risk aversion where, although the results are heterogeneous, there was a considerable amount of them, making it possible to more clearly show the implication of these polymorphisms in risk-taking. It is more difficult to draw conclusions with loss aversion because the results are heterogeneous, but also scarce (only five articles). This leads us to doubt whether the evidence found for this phenomenon is spurious or whether it really constitutes a good beginning to glimpse how loss aversion is modulated by genetic bases. Therefore, it is difficult to discuss if both phenomena are related and share genetic bases. Nonetheless, there are signs that make us think that this relationship could occur and that, with more research that addresses risk and loss aversion, this could be evidenced in a more robust way. In the following sections, we discuss the results for risk and loss aversion separately and in more detail.

### 4.1. Risk Aversion

#### 4.1.1. Risk Aversion and Dopaminergic Pathways

Starting with risk aversion and its relationship with dopaminergic pathways, we found the involved polymorphisms DRD4, MAOA, MAOA LPR, DAT1 and COMT Val^158^Met. All of them affect the level of dopamine that is available or the degree to which this neurotransmitter is able to act on the synaptic connections of the dopaminergic pathways, and especially on the striatum and the prefrontal cortex [55,59,60]. According to the results, many indicated that those alleles of the different polymorphisms whose presence translates into a lower level of dopamine or its decreased effectiveness of binding with receptors are associated with the lowest level of risk aversion. Thus, 7R+ allele carriers of DRD4 polymorphism, which tend to present some resistance in the dopamine-receptor binding [18], show the lowest risk aversion according to Kuhnen & Chiao [30]. The same was pointed out by Dreber et al. [18], although only in healthy men, highlighting the importance of gender differences when dealing with this phenomenon, something that has been pointed out in other behavioral studies, where men seem more likely to assume risks than women [61,62]. Lower risk aversion was also related to the low-active allele of MAOA [47] and the short allele of MAOA LPR [54], which, in turn, are related to the lower encoding and activity of the MAOA enzyme. This enzyme is one of the main contributors to dopamine metabolism [49]. Regarding MAOA LPR, it must be noted that behavioral differences between alleles were not found in risk-taking per se, but, more indirectly, in latency and delaying more in avoiding risk, which would indicate less risk aversion automatism. On the other hand, the 10R allele of DAT1 polymorphism, associated with the higher expression of dopamine transporters in the striatum, capable of recapturing this neurotransmitter and reducing the levels available in the synapse [51], was also related to lower risk aversion [51]. The same was observed in Zhong et al. [29], with the highest level of dopamine transporters associated with the lowest risk aversion. However, in this study, it was considered that it is the 9Rs (and not the 10Rs) which presents the highest level of transporters. This discrepancy may be due to the fact that, although it seems that 9R allele is associated with the greater final dopamine availability, there was a debate about the polymorphism, and studies could be found that defend both the first relationship and the second one, and even the non-relationship between the alleles of DAT1 and the levels of dopamine [51]. Despite this, the most important is that the lowest levels of dopamine in the synaptic cleft would be those reporting the lowest risk aversion, irrespective of whether it is caused by the 9R allele or the 10R allele. Finally, in relation to the COMT Val^158^Met, the Val allele was associated with lower dopamine levels due to the higher activity of the COMT enzyme, which is involved in the degradation of the neurotransmitter [42]. Thus, the carriers of this allele showed a reduced framing effect, which, in turn, could be explained as a smaller difference between risk-taking in loss contexts versus gain contexts due to the reduction in risk aversion in the latter context. Gao et al. [42] found that this relation between polymorphism and framing effect was mediated by the resting-state functional connectivity between orbitofrontal cortex and bilateral amygdala.

To date, we have highlighted only the studies that pointed to the same direction, but it is also possible to find several studies that found no relationship between each of the mentioned polymorphisms and risk aversion (for DRD4: [46,47,49,52,55]; for MAOA: [49], and for DAT1 and COMT Val^158^Met: [42]), as well as Amstadter et al. [48], which could even contradict the previous relationship reported for COMT polymorphism Val^158^Met. Finally, there was also a study [18] that investigated another polymorphism that is not mentioned above, DRD2, which affects the expression of the dopaminergic receptor D2, although no relationship with risk aversion was found. These inconsistencies could be due to several limitations. Most of the studies included in the review did not consider that the relationship between genes and risk aversion should consider all those phenotypes that could somehow influence this relationship. Thus, they tried to directly analyze whether having one allele or another presented different levels of risk aversion, but very few controlled whether other variables that could moderate or mediate this relationship. We have already seen how, for example, sex can be important. Other specific factors, such as suffering from a mental disorder (especially those linked to the reward system, e.g., RDS), age or personality, among many others, can also be important. Unfortunately, most of the studies (even those whose results were in the same direction) only considered the selection of a healthy sample without a history of pathologies and diseases and without the use of medication or drugs. In fact, some did not even specify that this selection was made [29,30,45,47,49,52,55] or, worse, they specified that their sample included some participants with disorders but did not address their influence on the analyses (e.g., [58]). Only one study [54] measured impulsivity and aggressiveness traits and controlled for them as possible confounding variables in the extraction of their results. Additionally, the specific characteristics of the tasks used may be relevant and should be considered in further studies. A gamble where, for example, you can gain vs. gain a lower amount is not the same as a gamble where you can gain vs. lose. In the absence of homogeneous contexts, results should be viewed with caution.

Given these limitations, it is difficult to know whether the relationship that the studies shown between genes and risk aversion really exists or was influenced by uncontrolled factors. However, this relationship would find theoretical support if we consider the known implication of dopamine in the brain reward system [34,35] and the connection that this system has with risk aversion. The dopaminergic pathways arise from the brainstem, from the ventral tegmental area (VTA) and the substantia nigra, and project towards various brain regions, mainly the prefrontal cortex and the striatum complex [63]. More specifically, the tegmental–ventral route, which runs from VTA to the ventral region of the striatum, and especially to the nucleus accumbens, seems to be the most important in relation to the processing of rewards [18,64]. Receiving a reward or anticipating it would increase dopamine levels in this pathway, which translates into increased physiological activation and the sensation of pleasure [65]. This dopamine elevation is reinforcing and is associated with approach behaviors [18], increasing the probabilities of performing the behavior that generates the neurotransmitter elevation. Given that the anticipation of an economic gain in a bet can produce such an increase, the more rewarding the profit is perceived to be and, therefore, the greater this increase, the more likely to assume risk in the bet [18,55]. In this line, those people who start from lower levels of dopamine or who are less sensitive to it (e.g., because they are carriers of some of the alleles that favor these effects, such as the 7R of DRD4 or 9R in DAT1), will require more intense stimulation to obtain the same pleasant effect that the rest of people would obtain with less stimulation [55].

This may explain the increased risk-taking associated with different alleles that, in one way or another, decrease the effects of dopamine. In fact, studies with rats show that those with higher levels of dopamine transporters show more impulsivity towards small rewards [66] or in humans, for example, the 7R+ allele of DRD4 has been linked to various risk behaviors such as alcoholism [67], impulsivity [68], sexual promiscuity and even infidelities [69]. Complementary evidence would come from neuroimaging studies searching for the neural bases of risk-taking, finding a positive correlation between the activity of the ventral striatum and the nucleus accumbens and the size of the possible reward [70,71] or those studies that specifically explore the bases of risk-aversion and also highlight the role of the ventral striatum in the search for reward, in interaction with the anterior cingulate cortex (ACC) and the inferior frontal gyrus, which seem to be activated proportionally to the risk that the situation entails [17]. As we can see, the mentioned regions are involved in dopaminergic pathways, reinforcing their involvement in risk aversion.

#### 4.1.2. Risk Aversion and Serotoninergic Pathways

On the other hand, the relationship between risk aversion and the serotonergic system must be considered. Here, we found three polymorphisms to be involved, the previously mentioned MAOA and MAOA LPR (since the enzyme MAOA is also involved in the metabolism of serotonin in addition to dopamine) and the 5-HTTLPR polymorphism. Regarding MAOA, the results were scarce and have already been discussed when talking about dopaminergic pathways. Thus, the main evidence for the involvement of serotonergic pathways in risk-aversion comes from studies focusing on 5-HTTLPR. In general, the short allele of this polymorphism is related to the lower transcription or lower activity of the serotonin transporters. As a result, serotonin transporters re-uptake less neurotransmitter, which leads to the higher availability of the neurotransmitter in the synapses [33]. Most studies suggest that the higher levels of serotonin linked to the short allele were associated with higher levels of risk aversion and susceptibility to framing effect, as indicated by Crişan et al. [33]. However, this relationship was not free of nuances either. As Heitland et al. [50] noted, the association between the short allele and the largest risk aversion was only in the gain contexts and not in loss contexts. This could be explained by the framing effect itself, given that, in loss contexts it would be more common to find risk-seeking and perhaps more difficult to find differences between the short and the long allele. However, the study was only conducted with women, which may reflect sex differences that explain this finding. Kuhnen & Chiao [30] pointed out that it was not enough to carry the short allele, but it was necessary to be homozygous s/s (two short alleles) to show greater risk aversion in both gain and loss contexts. Or Roiser et al. [43], who pointed out to the same need (to be homozygous), but this time to show greater susceptibility to framing effect, accompanied by increased amygdalin activity. It should be noted that there were also three studies that found no relationship between the alleles of 5-HTTLPR and risk aversion [44,47,52], and one [56], which could indicate an opposite result, reporting that s/s carriers show more risk-seeking, although only in contexts of loss, which could perhaps be explained by the framing-effect. Again, these inconsistencies may be due to the same reasons as those stated above. From the studies addressing 5-HTTLPR polymorphism, only four [33,43,50,56] ensured that a sample selection was free of disorders or conditions that might confound the results and only two [33,43] addressed other potentially influential factors, such as personality traits, anxiety, or impulsivity. However, only one [43] actually took these factors into account and controlled for them in the analyses when extracting results.

As in the dopaminergic pathway, these limitations make it difficult to draw firm conclusions; however, it seems evident that the serotonergic pathway and the genes that regulate it are somehow involved in the risk-aversion phenomenon. Some previous examples, but not including the genetic part, also suggest this: it seems that the elevated levels of serotonin after the administration of its precursor (tryptophan) or inhibitors of its transporters, facilitate the recognition of threatening faces in humans [72,73], and the acquisition of a conditioned fear in rats [74]; however, the depletion of tryptophan impairs the recognition of threatening faces [75] and the distinction between gain and loss contexts when making decisions [76]. Again, this would make sense if we consider that serotonin is a key neurotransmitter in the regulation of emotional processes [33] and that risk aversion seems to be closely related to, or at least influenced by, the emotions [9]. In fact, positive correlations have been found between negative emotions such as fear, anger or sadness and the level of risk aversion [60,77]. In addition, ACC, one of the neural bases of risk aversion [17], has been highlighted as an important region in emotional processing [78,79], which can modulate amygdala activity [80] and serve as a connection between the limbic system and the prefrontal cortex [81]. Finally, Shiv et al. [82] showed that patients with lesions in regions such as the amygdala or insula, which are closely related to emotions, showed less risk aversion than other patients with injuries in other regions not related to emotions or healthy controls. All this leads us to think that, if certain genes influence the availability of serotonin in the brain and this, in turn, can influence the brain’s own activity (in this case, conditioning the capacity for emotional processing and regulation), behaviors such as risk aversion, which depend, at least partially, on this activity, would ultimately be affected. 

The connection between genes and behavior does not seem to be direct but moderated by the final availability of neurotransmitters and by the resulting neural activity. However, most studies included in this review were looking for the relationship between genes and behavior-skipping intermediaries. This can be an important source of heterogeneity in the results, since there would be many factors (e.g., sex, age, or stress, among others), and not only carried a gene allele, which can influence the levels of neurotransmission and neural activity. Only two articles [44,54] studied genetics in parallel with the neural activity, holding that the relationship between polymorphisms and risk-aversion was mediated by the activation of certain regions, such as the amygdala or the orbitofrontal cortex. It would be interesting to use a similar approach in the future to better address the complexity of these relationships.

Based on the above, we could conclude that risk aversion is closely linked to emotions. The genes highlighted in our review are those that directly impact on dopaminergic and serotonergic neurotransmission, pathways that are intimately linked to emotion expression and regulation. Therefore, our results support a relative implication of the dopaminergic and serotonergic neurotransmission pathways in risk-aversion. Further research addressing the modulating or mediating role of other possible confounding variables is needed. In addition, future studies will also need to consider the emotional nature of risk aversion and how it may affect even more complex economic behaviors. For example, the recent study by Liu et al. [83] assesses how to resolve difficulties in carrying out crowdfunding projects when the context is adverse, such as that arising from the COVID-19 pandemic, and points to the importance of eliminating participants’ distrust and concerns. Knowing that risk aversion has an emotional origin, it could be addressed through affective regulation programs that promote more rational decision-making and facilitate people’s involvement in such projects. Similarly, risk aversion should be considered when analyzing other macroeconomic aspects, such as the reduction of a country’s debt [84] or the digitalization of currency [85], as these macroeconomic processes involve microeconomic behaviors, carried out by people who, in turn, are affected by emotional phenomena such as risk aversion.

### 4.2. Loss Aversion

Although loss aversion has been one of the most studied phenomena in social sciences since it was described, articles investigating its genetic basis were scarce, with only five studies being found, which presented a high heterogeneity.

Regarding serotonergic pathways, 5-HTTLPR polymorphism was addressed in four studies. In He et al. [31], homozygous for s/s (but not s/l or l/l carriers), presented greater loss aversion, in line with what occurred with risk aversion. However, in Anderson et al. [32], Neukam et al. [56] and Ernst et al. [57], this relationship was not confirmed. The latter also included a group of patients with anxiety disorder in which being homozygous for allele l/l was associated with lower levels of loss aversion, evidence that could be complementary to that of the study by He et al. [31]. This result again emphasizes how important is to avoid studying the relationship between polymorphisms and behavior in isolation from other aspects. The assessment of contextual and organic aspects, as in this case, the presence of pathologies or age, seems to be crucial. As with gender differences, these factors could modulate the relationship between genes and behavior [57], for example, by altering neurotransmission levels in other ways. In fact, following this example, there is also a relationship between anxiety and the genes that influence the serotonergic system [58,86]. Given that the polymorphisms seem to influence behaviors such as loss aversion through their influence on neurotransmission pathways, it is important to address the variables that also influence these pathways. This consideration would be extended to risk aversion. We can turn again to the study by Eisenegger et al. [46], where a priori relations between DRD4 polymorphism and risk aversion were not found, but manifested after the administration of a dopamine dose.

On the other hand, with regard to the dopaminergic pathways and loss aversion, only DRD4 and DRD2 polymorphisms were studied and none of their alleles were related to the level of loss aversion [32,52]. However, when DRD2 was studied in interaction with another polymorphism, BDNF Val^66^Met, it shown remarkable results [32]. Specifically, the A1 allele of DRD2, which is associated with a lower dopamine-receptor junction, was related to the lower level of loss aversion. This is like what we described in risk aversion, where lower sensitivity to dopamine led to more risks, but only when it was combined with the Met allele of BDNF Val^66^Met, associated with lower BDNF production [32]. This reaffirms the complexity of the relationship between genetics and behavior, as well as the need of not studying polymorphisms in isolation. As Harrati [26] and Gao et al. [53] argued, studying polymorphisms in candidate genes constitutes an advantage with respect to the study of SNPs, given that this poses “handfuls rather than millions of statistical significance tests at stake, so much weaker associations can be found to pass thresholds for statistical significance” [26], p. 187. However, when complex behaviors such as risk or loss aversion are involved, they are most likely related to multiple genetic variants at the same time [41,53] and studying just one polymorphism may not be enough. The study by Gao et al. [53] followed a similar approach to that of Harrati [26], where millions of SNPs were taken and their relationship with behavior was analyzed (without showing significant results); however, on this occasion, addressing multiple polymorphisms and not SNPs. Specifically, the study analyzed polymorphisms of 26 different genes, and thus could approach a behavior from a broader genetic approach and overcome many of the limitations that the rest of the studies presented. With this approach, Gao et al. [53] pointed out that genes such as SLC6A4, COMT and DDC, which are involved in the serotonergic and dopaminergic pathways, would partially explain the individual differences in framing effect. However, approaches such as this have not been found to date in studies of loss aversion.

As we can see, the results for loss aversion were scarce and heterogeneous, so it is difficult to draw a conclusion about its possible genetic basis or its relationship to risk aversion. This leads us to think that it may be too early to answer this question and more research is needed. Nevertheless, the scarce evidence subtly points, as in risk aversion, to the implication of genes involved in dopaminergic and serotoninergic neurotransmission. Previously, loss aversion had also been linked to these pathways, since its neural basis indicates the involvement, again, of the brain’s emotional system [15], with the amygdala, the insula, the ACC, the striatum and the prefrontal cortex being the main regions involved. In addition, some studies addressed how different emotional regulation strategies were able to reduce loss aversion [13,87], how patients with injuries in regions typically identified as emotional lacked this bias [88], or how, certain factors such as unpleasant odors, which are capable of inducing a negative emotional state, increased loss aversion [89]. This suggests that if more studies were carried out in the genetic field, the implication of a solid genetic bases, probably related to the dopaminergic and serotoninergic pathways, could be found. With this, the relationship between risk aversion and loss aversion could be more clearly demonstrated in the future.

### 4.3. Limitations and Future Research

As we have seen, our review is influenced by several limitations. Thus, part of the heterogeneity found in the results could derive from methodological aspects. For risk aversion, although we have limited the inclusion of studies to a specific type of tasks (investment task, gambling task and BART), these can still present variability: changing the amounts at stake, waiting times, and the way in which bets are presented, among other differences. Likewise, there is also debate about whether to measure risk-taking with tasks or with questionnaires, “ask or task”. Questionnaires provide more stable measures (and probably more linked to genetics), while tasks seem to be more affected by contextual aspects at the time of the experiment [90]. Additionally, since its origin, risk aversion has been studied using similar tasks (gambling tasks), but some involved the possibility of losing and others did not. Thus, some contrast safe gains with the risk of (if heads) winning more or (if tails) losing (e.g., [30]), but others contrast safe gains against the risk of (if heads) winning more or (if tails) winning less than the safe gain (e.g., [91]). The former involves risk of loss and, although they are commonly used to measure risk aversion, it is difficult to determine whether their measurement does not rather reflect the level of loss aversion. Unfortunately, most gambling tasks used in this review included the possibility of losing in their protocols. This is another important limitation that prevents us from knowing if the similarity between the results found in risk and loss aversion comes from the common origin of these biases or because the tasks used measure all loss aversion and not risk aversion.

Another important source of heterogeneity could come from the samples used. Thus, as previously mentioned, it is important (both in risk and loss aversion) to focus on homogeneous samples and to deal more specifically with all the possible factors that could mediate or modulate the genes–behavior relationship, an aspect that most of the studies lacked. This is a key point and, because of this, it is difficult to determine whether current results are valid or may be subject to uncontrolled factors. In addition, many sample sizes did not exceed one hundred participants (e.g., [33,43]), making it difficult to obtain firm results given the small size of the effect associated with each polymorphism separately [41,53].

Finally, as can be seen from the heterogeneity of the results, and given that they have been published in journals from very different areas (e.g., economics or biology), the results do not seem to be particularly affected by the publication bias that favors the publication of positive results. However, given that our approach to risk and loss aversion is framed in the economic field, we must warn of the presence of a specific bias stemming from this.

In order to overcome the present limitations of this review, and improve the quality of future studies, they should follow these recommendations:

1. In line with Gao et al. [53], since risk and loss aversion are complex behaviors, studies should address multiple candidate genes at once. In addition, they should be open to testing other genes outside the dopaminergic and serotonergic pathways to avoid confirmation bias.
2. Sufficient samples should be included to ensure that the multiple contrasts required have adequate power. More samples imply more effort, but only then will the studies be valuable.
3. It is necessary to control all possible confusing variables that could alter the genes–behavior relationship. Other behavioral and health measurements should be provided, but it would also be useful to rely on neuroimaging techniques and other means to measure or infer the level of neurotransmitters that is available.
4. It should be checked whether the relationship between genes and risk aversion is affected depending on whether tasks or questionnaires are used.
5. To discriminate between risk and loss aversion, it would be advisable to use only tasks whose risk does not include losses.

## 5. Conclusions

As far as risk aversion is concerned, the studies are heterogeneous, but most of them highlight the polymorphisms involved in dopaminergic and serotonergic neurotransmission pathways. These polymorphisms are related to higher or lower levels of risk aversion depending on the allele they adopt. This supports previous evidence connecting risk aversion to emotions, since the latter are intimately linked to these dopaminergic and serotoninergic pathways. On the other hand, regarding loss aversion, it is too early to draw conclusions and this review should only be taken as a starting point for future studies investigating the genetic basis of loss aversion and its connection with risk aversion. Those studies should follow the recommendations provided in this study to avoid falling back into the same limitations that prevent us from shedding more light on this study field. Whether risk aversion and loss aversion share a common emotional origin is a question that needs to be answered with further research.

## Figures and Tables

**Table 1:** Risk Aversion.

Authors	Sample	Genetical Measures	Health & Behavioral Profile	Risk Aversion Measures	Results
Crişan et al. [33]	32 participants (23 women, *M* = 26.75 years, *SD* = 6.69)	Genotyping for the 5-HTTLPR: s/s, s/l and l/l	Healthy volunteers *. s-carriers show higher anxiety trait. No other behavioral variables were reported	Risk-taking with BART; Framing effect with a gambling task	The s-carriers showed higher risk aversion and framing effect than l-homozygotes, without controlling for anxiety trait
Khunen and Chiao, [30]	65 participants (48 women, *M* = 22.4 years, *SD* = 4.9)	Genotyping for the 5-HTTLPR: s/s, s/l and l/l; and for the DRD4: 7R+ vs. 7R− allele	No health or behavioral variables were reported	Risk-taking with investment task	s/s homozygotes took less risk than s/l or l/l-; 7R+ carriers took more risk than 7R− carriers
Roe et al. [42]	67 participants (29 women, *M* = 20.6 years, *SD* = 3.2)	Genotyping for COMT Val^158^Met: Met/Met, Met/Val and Val/Val	55 healthy volunteers * and 12 participants diagnosed with depression, bipolar disorder or another. No other behavioral variables were reported	Risk attitudes with a gambling task	Risk attitudes were not associated with COMT polymorphism. Analysis did not control for the pathological conditions
Roiser et al. [43]	30 participants	Genotyping for the 5-HTTLPR: s/s and l/l	Healthy volunteers *. Personality, impulsiveness and state-trait anxiety were not reported, but controlled in further analysis	Framing effect with a gambling task	The s/s genotype group exhibited a greater amygdala activity and framing effect while making choices than s/l or l/l genotype (controlling for behavioral and health variables)
Zhong et al. [44]	350 participants (188 women, *M* = 28.2, *SD* = 10.8)	Genotyping for the 5-HTTLPR: s/s, s/l and l/l; and for the DAT1: 9R vs. 10R allele	No health or behavioral variables were reported	Risk attitudes with multiple price list design	l-carriers of 5-HTTLPR tended to be (not significant) more risk-tolerant over losses than s-carriers; 9R carriers of DAT1 were more risk-tolerant over gains than 10R
Dreber et al. [18]	98 male participants (ranging from 18 to 23 years)	Genotyping for the DRD4: 7R+ vs. 7R− allele; and for the DRD2 Taq1a/ANKK1: A1+ vs. A1-	Healthy volunteers *. No other behavioral or health variables were reported	Risk preferences with an investment task	No associations were found between the A1+ carriers and risk preferences. 7R+ carriers were more risk loving than 7R−
Dreber et al. [45]	237 participants	Genotyping for the DRD4: 7R+ vs. 7R− allele	No health or behavioral variables were reported	Risk-taking in the card game bridge, and an investment task	7R+ men showed higher risk-taking in bridge and investment task than 7R−. No effects in women
Eisenegger et al. [46]	205 male participants (*M* = 23.5 years, *SD* = 3.6)	Genotyping for the DRD4: 7R+ vs. 7R− allele	Healthy volunteers *. No other behavioral or health variables were reported	Risk-taking with a gambling task	No relation between genotype and risk-taking was found directly, but 7R+ carriers showed an increased gambling propensity after dopaminergic stimulation
Frydman et al. [47]	83 male participants	Genotyping for the 5-HTTLPR: s/s, s/l and l/l; for the DRD4: 7R+ vs. 7R− allele; and for the MAOA: MAOA-H vs. MAOA-L	No health or behavioral variables were reported	Risk-taking with a gambling task	MAOA-L carriers were more likely to take financial risks. No differences among the 5-HTTLPR and DRD4 polymorphisms
Amstadter et al. [48]	223 children (44.4% female, *M* = 11.3 years)	Genotyping for the COMT Val^158^Met: Met/Met, Met/Val and Val/Val	Healthy * volunteers. No other behavioral or health variables were reported	Risk-taking with BART	Females Met-carriers, but not males, showed higher risk taking compared to Val homozygotes
Dreber et al. [49]	135 participants (women 8%, median age was 43 years)	Genotyping for the DRD4: 7R+ vs. 7R− allele; and for MAOA gene: MAOA-H vs. MAOA-L	No health or behavioral variables were reported	Risk-taking with an investment task	No significant relations were found between genes and risk-taking
Heitland et al. [50]	60 females (*M* = 20.87 years, *SD* = 1.98)	Genotyping for the DAT1: 9R+ vs. 9R− allele; for the COMT Val^158^Met: Met/Met, Met/Val and Val/Val; and for the 5-HTTLPR: s/s, s/l and l/l	Healthy volunteers *. No other behavioral or health variables were reported	Risk-taking with a gambling task	DAT1 9R+ allele showed a trend toward increased risk-taking following losses (no significant effect). Genotypes of COMT did not show any relation with risk-taking. 5-HTTLPR s-carriers showed decreased risk-taking following gains.
Mata et al. [51]	322 participants (234 women; *M* = 23.8 years, *SD* = 6.2)	Genotyping for the DAT1: 9R vs. 10R allele	Healthy volunteers *. No other behavioral or health variables were reported	Risk-taking with BART	10R allele showed increased risk-taking respect to 9R
Harrati, [26]	10,455 adults	Over 2.5 million Single Nucleotide Polymorphisms (SNPs) from respondents	No health or behavioral variables were reported	Risk aversion through responses to a series of hypothetical gambles on lifetime income from HRS	None of the single-nucleotide polymorphisms were found to be determinants of risk aversion
Anderson et al. [52]	174 participants	Genotyping for the 5-HTTLPR polymorphism: s/s, s/l and l/l; and for the DRD4 gene: 7R+ vs. 7R− allele	No health or behavioral variables were reported	Risk-taking with a multiple price listing	No significant correlations between the two genes and risk-taking
Gao et al. [53]	111 students (36% women, *M* = 21.78 years, *SD* = 61.92)	Genotyping for the COMT Val^158^Met: Met/Met, Met/Val and Val/Val	Healthy volunteers *. No other behavioral or health variables were reported	Framing effect with a gambling task	The Met-carriers showed greater framing effect than Val/Val homozygotes and this was mediated by resting-connectivity between orbitofrontal cortex and bilateral amygdala
Gao et al. [42]	1582 students (80.1% women, *M* = 18.66 years, *SD* = 60.90)	Gene-based approach considering 26 genes from the serotoninergic and dopaminergic pathways	Healthy volunteers *. Normal range of depressive and anxiety symptoms. No other behavioral or health variables were reported	Framing effect with a gambling task	Genetic variations of the SLC6A4, the COMT and DDC genes were associated with the framing-effect
Wagels et al. [54]	105 male participants	Genotyping for the MAOA LPR: MAOA-S vs. MAOA-L	Healthy volunteers *. Anxiety and aggressiveness were not reported but controlled in further analysis. No other behavioral or health variables were reported	Risk-taking with BART	MAOA s-carriers showed less automatic harm avoidance, but no differences were found in BART. MAOA s-carriers were more risk-taking after testosterone administration. These results were found controlling for anxiety and aggressiveness
Muda et al. [55]	113 investors, *M* = 33.70 years, *SD* = 9.95 & 104 non-investors, *M* = 32.34, *SD* = 10.00)	Genotyping for the DRD4: 7R+ vs. 7R− allele	No health or behavioral variables were reported	Risk-taking with a gambling task	No differences in risk-taking between 7R+ and 7R− individuals
Neukam et al. [56]	577 participants	Genotyping for the 5-HTTLPR: s/s and l/l	Healthy volunteers *. No other behavioral or health variables were reported	Risk-taking with Value-based decision-making battery	s/s homozygotes were more risk-seeking for losses compared to s/l and l/l

*M*, mean; *SD*, standard deviation; BART, Balloon analogue risk task. * These participants were screened for no history of neuropsychiatric or chronic somatic conditions (including substance abuse disorder and pathological gambling) and no medication or drug consumption.

**Table 2:** Loss aversion.

Authors	Sample	Genetical Measures	Health & Behavioral Profile	Loss Aversion Measures	Results
He et al. [31]	572 participants (312 females, *M* = 20.47 years, *SD* = 1.01)	Genotyping for the 5-HTTLPR: s/s, s/l and l/l	Healthy volunteers *. No other behavioral or health variables were reported	Loss aversion with a mixed gamble task	s/s individuals had higher loss aversion and these effects were stronger for males than females
Ernst et al. [57]	66 adolescents	Genotyping for the 5-HTTLPR: s/s, s/l and l/l	27 participants with (only) anxiety disorder and 39 healthy *. No other behavioral or health variables were reported.	Loss aversion with a mixed gamble task	No differences between genotypes in healthy controls. Lower loss aversion in l/l anxious individuals. No effect in s/s anxious adolescents
Anderson et al. [52]	174 participants	Genotyping for the 5-HTTLPR: s/s, s/l and l/l; and for the DRD4: 7R+ vs. 7R− allele	No health or behavioral variables were reported	Loss aversion with hypothetical choices in a survey	No significant correlations between the two genes and loss aversion
Voigt et al. [32]	143 participants (114 females, *M* = 21.8 years, *SD* = 4.04)	Genotyping for the BDNF Val^66^Met: Val/Val, Val/Met and Met/Met; and for the DRD2 Taq1a/ANKK1: A1A1, A1A2 and A2A2	No health or behavioral variables were reported	Loss aversion with a mixed gamble task	Significant interaction of the 2 polymorphisms: carriers of the genetic constellation Met+/A1+ show the lowest loss aversion
Neukam et al. [56]	611 participants	Genotyping for the 5-HTTLPR: s/s, s/l and l/l	Healthy volunteers *. No other behavioral or health variables were reported	Loss aversion with a mixed gamble task	No significant results were found

*M*, mean; *SD*, standard deviation; BART, Balloon analogue risk task. * These participants were screened for no history of neuropsychiatric or chronic somatic conditions (including substance abuse disorder and pathological gambling) and no medication or drug consumption.
